# Postoperative Compensatory Ammonium Excretion Subsequent to Systemic Acidosis in Cardiac Patients

**DOI:** 10.1155/2017/5383574

**Published:** 2017-05-22

**Authors:** Friederike Roehrborn, Daniel-Sebastian Dohle, Indra N. Waack, Konstantinos Tsagakis, Heinz Jakob, Johanna K. Teloh

**Affiliations:** ^1^Institute of Physiological Chemistry, University Hospital Essen, University of Duisburg-Essen, Hufelandstrasse 55, Essen, 45147 North Rhine-Westphalia, Germany; ^2^Department of Thoracic and Cardiovascular Surgery, West German Heart Center, University Hospital Essen, Hufelandstrasse 55, Essen, 45147 North Rhine-Westphalia, Germany

## Abstract

**Background:**

Postoperative acid-base imbalances, usually acidosis, frequently occur after cardiac surgery. In most cases, the human body, not suffering from any severe preexisting illnesses regarding lung, liver, and kidney, is capable of transient compensation and final correction. The aim of this study was to correlate the appearance of postoperatively occurring acidosis with renal ammonium excretion.

**Materials and Methods:**

Between 07/2014 and 10/2014, a total of 25 consecutive patients scheduled for elective isolated coronary artery bypass grafting with cardiopulmonary bypass were enrolled in this prospective observational study. During the operative procedure and the first two postoperative days, blood gas analyses were carried out and urine samples collected. Urine samples were analyzed for the absolute amount of ammonium.

**Results:**

Of all patients, thirteen patients developed acidosis as an initial disturbance in the postoperative period: five of respiratory and eight of metabolic origin. Four patients with respiratory acidosis but none of those with metabolic acidosis subsequently developed a base excess > +2 mEq/L.

**Conclusion:**

Ammonium excretion correlated with the increase in base excess. The acidosis origin seems to have a large influence on renal compensation in terms of ammonium excretion and the possibility of an overcorrection.

## 1. Introduction

Nowadays, coronary artery bypass grafting (CABG) surgery is a routine procedure. In conventional on-pump CABG surgery procedures using crystalloid cardioplegic solution, dilutional acidosis is frequently found after administration of the cardiopulmonary bypass (CPB) priming volume and cardioplegia [1–4]. Lactic acidosis might also develop during the operative and postoperative course depending on different parameters like CPB and cross-clamp duration and postoperative hemodynamic status [1, 5, 6]. Nonetheless, acid-base imbalances are present not only during the operation but also commonly in the postoperative phase, which are then of all sorts, with acidosis prevailing [1, 7, 8]. Severe manifestation causes, among others, impairments of the cardiovascular system with concomitant systemic consequences [9–11]. Sometimes, those imbalances need correction during the patient's stay in the intensive care unit (ICU), whereas in most cases the human body, not suffering from any severe preexisting illnesses regarding lung, liver, and kidney, is capable of transient compensation and final correction. For this purpose, extracellular buffering, respiratory compensation, intracellular buffering, and renal compensation are available in general [9]. Regularly, patients show different postoperative imbalances soon following each other. Due to the frequent incidence of acidosis, instead of alkalosis, we only focused on those patients that suffered from initial respiratory or metabolic acidosis in the postoperative phase. To date, to the best of our knowledge, no data exist evaluating compensatory ammonium excretion in the context of postoperative acidosis of either origin after CABG and cardiac surgery in general. Therefore, we analyzed routine blood gas parameters of the early postoperative period, evaluated possible acid-base imbalances, and correlated these to the ammonium excretion quantitatively assessed in urine samples. We hypothesized that there is a correlation between the appearance of postoperatively occurring acidosis and renal ammonium excretion. The aim of this study was to investigate this hypothesis.

## 2. Materials and Methods

### 2.1. Study Design and Patient Population

As described previously, a total of 25 patients supposed to have CABG surgery were enrolled in a prospective nonrandomized observational study at the Department of Thoracic and Cardiovascular Surgery affiliated to the University Hospital Essen from July 2014 until October 2014 [4]. The study has been approved according to the guidelines of the Declaration of Helsinki by the Medical Ethics Committee of the University Hospital Essen. All patients gave their written informed consent. Patients with acute myocardial infarction with or without ST-interval elevation, cardiogenic shock, concomitant cardiac diseases and procedures, or already existing preoperative acidosis and those who participated in other studies were not considered for the study.

In general, urine samples from these patients undergoing CABG surgery were collected from the beginning of the procedure. They were obtained through an intraurethral catheter inserted immediately before the operation, which was kept in place for at least two days. The first sample was obtained shortly after catheterization. The second sample was drawn from the whole amount of urine in the catheter bag collected during the operation. The third sample was gathered from the whole amount in the catheter bag collected since the sampling at the end of the operation until 9 p.m. of the operation day. Further samples were collected every eight hours (5 a.m., 1 p.m., and 9 p.m.) for the first two postoperative days. The urine collected in every case was a mixture of all urine produced during the last collection period. The respective total urine volume having been excreted within the last eight hours was noted. Until analysis, the samples were stored at −80°C.

### 2.2. Ammonium Measurements

For determination of ammonium, capillary electrophoresis (P/ACE MDQ, Beckman & Coulter) was employed, which is known to constitute a highly efficient and effective separation method for analysis of ionic species in general and thus also ammonium in particular [12–15]. The capillary was made of* fused silica* and had an effective length of 50 cm with an inner diameter of 75 *µ*m and outer diameter of 375 *µ*m. The samples were diluted 50-fold with ultrapure water. If no ammonium could be detected using this dilution factor, measurement was repeated with a 10-fold dilution. The volume was injected into the capillary using pressure injection, and separation took place at 30 kV with normal capillary polarity. Measurements were executed using a Cation Analysis Kit (ABSciex, Darmstadt, Germany). Indirect detection was performed with the help of a diode array analyzer at 200 nm. The temperature of tray and capillary was set to 25°C. The measurements were performed by one person only who underwent a detailed initial training program previously.

The absolute amounts of ammonium were calculated by taking the total urine volume of which the sample was taken into consideration.

### 2.3. Acid-Base Status

Blood gas samples were routinely taken and analyzed (ABL800 FLEX, Radiometer GmbH, Willich, Germany) for hemoglobin, standard acid-base parameters (pH value, bicarbonate, and base excess), electrolytes (Na^+^, K^+^, Ca^2+^, and Cl^−^), glucose, and lactate. Moments of withdrawal were not controlled but were standardized by hospital practice (dependent on the patient individual need). Samples were taken by the well-trained intensive care nurse responsible for the respective patient. The blood gas analyzer was subjected to the internal quality management as regards maintenance but also daily calibration and measurement of special quality controls.

The reference range for carbon dioxide partial pressure was set to 35 mmHg to 45 mmHg and for base excess (BE) −2 mEq/L to +2 mEq/L. Imbalances were called acidosis or alkalosis, respectively, if at least three out of five consecutive blood gas analyses indicated a disturbance in one of the parameters. Patients were grouped according to their disturbances in acid-base status: initial postoperative respiratory acidosis and no subsequent BE > +2 mEq/L, initial postoperative respiratory acidosis and subsequent BE > +2 mEq/L, initial postoperative metabolic acidosis and no subsequent BE > +2 mEq/L, and initial postoperative metabolic disorder and subsequent BE > +2 mEq/L.

Postoperative BE values were determined as follows: after cessation of the initial individual acid-base disturbance in the postoperative course, all subsequent BE values from all patients within one group (see above) were used in the two-day observation period to calculate the mean value.

During the operation as well as in the postoperative phase, patients received different fluids intravenously (Table 1), according to those values published previously [4, 16].

Urinary ammonium excretion was evaluated after emergence of respiratory and metabolic acidosis, respectively, over the course of time as well. The eight-hour interval in which the largest acid-base imbalance occurred was determined for every patient individually on the basis of his/her values. The ammonium value of this interval (“disturbance”) was taken and the mean was compared to the mean of ammonium values from the subsequent eight-hour interval (“compensation”).

### 2.4. Statistical Analysis

The data are expressed as mean values ± standard deviation. Analysis of the contingency table was performed by using Fisher's exact test. Comparison of two groups was realized by using an unpaired *t*-test with Welch's correction. A *p* value of at least *p* < 0.05 was considered significant.

## 3. Results

**Figure 1:**
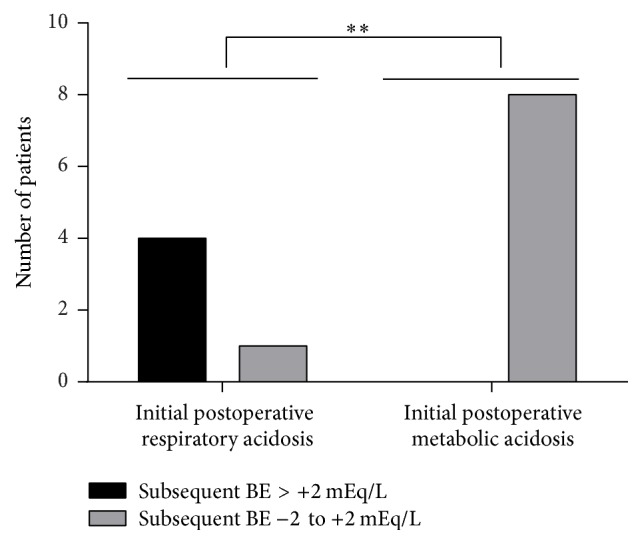
Development of a subsequent disturbance with a base excess > +2 mEq/L dependent on the initial postoperative type of acidosis (respiratory versus metabolic) in patients scheduled for elective isolated coronary artery bypass grafting. ^*∗∗*^*p* < 0.01.

After analysis of plasma acid-base status, five patients were not further analyzed because they did not develop any postoperative acid-base disturbances. Furthermore, two patients were not analyzed, because they primarily presented with alkalosis. Five patients were not taken into further consideration because they displayed either combined (respiratory as well as metabolic) disturbances or one disturbance shortly following another. This led to a final population of thirteen patients developing acidosis of either but only of a single origin as an initial disturbance in the postoperative period. Of these thirteen patients, five developed a condition with carbon dioxide partial pressure >45 mmHg and eight had a BE < −2 mEq/L. Four out of five patients initially displaying respiratory acidosis developed a BE > +2 mEq/L subsequently, whereas one did not develop any subsequent disturbance. Those eight patients developing metabolic acidosis at first in no case exhibited a BE > +2 mEq/L subsequently (Figure 1).

**Figure 2:**
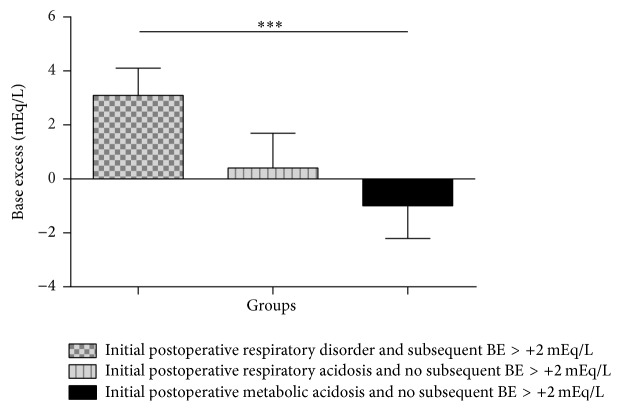
Mean base excess value of the patients grouped according to their acid-base status in the postoperative phase. Groups were initial postoperative respiratory acidosis and no subsequent BE > +2 mEq/L, initial postoperative respiratory acidosis and subsequent BE > +2 mEq/L, initial postoperative metabolic acidosis and no subsequent BE > +2 mEq/L, and initial postoperative metabolic disorder and subsequent BE > +2 mEq/L. Postoperative BE values were determined as follows: after cessation of the initial individual acid-base disturbance in the postoperative course, all subsequent BE values from all patients within one group (see above) were used in the two-day observation period to calculate the mean value. Values are shown as mean ± standard deviation. ^*∗∗∗*^*p* < 0.001.

Of the patients having initially presented with respiratory acidosis in the postoperative course and subsequently developed BE > + 2 mEq/L, the mean BE was 3.1 ± 1.0 mEq/L (Figure 2). The patient with initial postoperative respiratory acidosis and no subsequent metabolic imbalance showed a BE of 0.4 ± 1.3 mEq/L over the course of time. Patients with initial postoperative metabolic acidosis who did not show subsequent acid-base disturbances displayed a mean BE of −1.0 ± 1.2 mEq/L. No patients showed metabolic acidosis in the postoperative course at first and subsequently a BE > +2 mEq/L.

**Figure 3:**
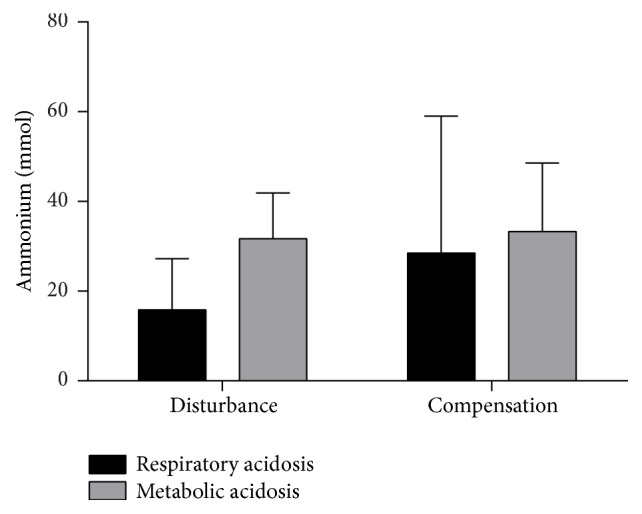
Ammonium excretion as a function of the initial disturbance in acid-base status (respiratory versus metabolic acidosis) over the course of time. Urine samples were collected every eight hours from the entire volume that had been excreted during this interval. With the help of the blood gas analyses, the interval with the largest deviation from the reference value (base excess and carbon dioxide partial pressure, resp.) was determined and, thus, the ammonium value from the respective interval (“disturbance”). Accordingly, the ammonium value from the subsequent interval was determined (“compensation”) in order to evaluate time dependence. For patients suffering from respiratory acidosis, but not for those from metabolic acidosis, there is a trend in rising urinary ammonium values in the eight-hour interval subsequent to the acid-base imbalance. This may lead to the observed overcorrection. Values are shown as mean ± standard deviation.

During the eight-hour interval in which the largest deviation from the reference value (BE and carbon dioxide partial pressure, resp., depending on the type of acidosis) was measured, mean urinary ammonium was 15.8 ± 11.4 mmol for patients with initial postoperative respiratory acidosis and 31.7 ± 10.2 mmol for those with initial postoperative metabolic acidosis (Figure 3). The urinary ammonium value of the subsequent eight-hour interval increased to 28.5 ± 30.5 mmol and 33.3 ± 15.3 mmol, respectively.

**Figure 4:**
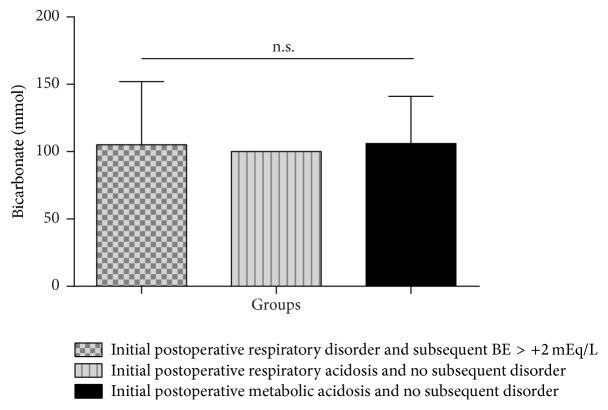
Intraoperative amounts of administered sodium bicarbonate. In the context of intraoperative acid-base status management, all patients received sodium bicarbonate by the perfusionist to antagonize dilutional acidosis originating from introduction of the priming and the cardioplegic solution into the patients' systemic circulation, which are both devoid of bicarbonate. Values are shown as mean ± standard deviation. n.s. = not significant.

Patients having initially presented with respiratory acidosis in the postoperative course and subsequently developed BE > +2 mEq/L received 105 ± 47 mmol bicarbonate in the intraoperative interval (Figure 4, Table 1). The patient with initial postoperative respiratory acidosis and no subsequent metabolic imbalance received 100 mmol bicarbonate. Patients with initial postoperative metabolic acidosis who did not show subsequent acid-base disturbances received 106 ± 35 mmol bicarbonate.

## 4. Discussion

The main result of the present observational study is that none of the patients having initially suffered from metabolic acidosis in the postoperative course subsequently developed a BE > +2 mEq/L, whereas 80% of patients having had initial respiratory acidosis showed hereinafter a BE > +2 mEq/L (Figure 1).

Extracellular metabolic acidosis influences different parameters and organ systems of the organism due to its inherent properties. Among others, the heart's contractility is diminished, peripheral vasodilatation is enhanced, both resulting in decreased blood pressure, the responsiveness to catecholamines is reduced, and the risk of arrhythmias is increased [17, 18]. Thus, the human body in general possesses four mechanisms for compensation: extracellular buffering, respiratory compensation, intracellular buffering, and renal compensation [9]. After generation of excess extracellular protons, they are immediately bound by extracellular buffers like bicarbonate, proteins, and phosphate. The second possibility for compensation constitutes adapted respiration which is hyperventilation in the case of acidosis. Respiratory compensation is also realized very quickly. During the process of intracellular buffering, protons are transported into cells in exchange for other monovalent cations like sodium or potassium. Inside the cell, these protons bind to common proteins or hemoglobin. Probably, this mechanism is also rapidly instating. Nevertheless, it serves the purpose of only transient neutralization; the exchange of cations is continuously reversed in the later course. The fourth possibility is renal compensation which in turn can be divided into three mechanisms: excretion of free protons (accounting for urine acidification), excretion of phosphate, and excretion of ammonium [19]. Since the pH of urine is physiologically restricted between 4.5 and 7.5, only small amounts of H^+^ can be excreted this way (<0.05 mM at maximum) [20–22], whereas the phosphate excretion under physiologic conditions is considerable (~25 mmol/day) [23, 24]. However, attributable to the acidification of urine, hydrogen phosphate being present in the urine is further protonated to yield dihydrogenphosphate. However, this buffer system is barely gradable due to limited available phosphate (doubling or tripling at maximum) [21, 22, 24, 25]. Regarding these renal mechanisms, ammonium excretion is the dominant variable under physiologic conditions as well as in acidosis [26, 27]. According to common literature, ammonium excretion increases within a few hours after acidosis induction and thus is the primary component of the increase in net acid excretion [19, 24, 28–31] which was proven in several experimental animal studies. All of these studies employed either hydrochloric acid or ammonium chloride for acidosis induction. Nevertheless, the transferability of these results to clinical relevant forms of acidosis is given as was demonstrated in an experimental model of normovolemic hemodilution (Teloh et al., accepted). In accordance with the experimental protocol, rats were stepwise hemodiluted to a systemic hematocrit of 10% which favored the development of dilutional acute metabolic acidosis, principally comparable to cardiosurgical patients. Since excreted ammonium derives from deamination reactions, glutaminase I and glutamate dehydrogenase are required [32–34]. In acidotic states, urinary ammonium excretion rises, resulting from interplay between renal glutamine degradation with a concomitant fall in glutamine concentration and increased ammonium excretion due to urinary acidification. In the liver, resynthesis of glutamine occurs for disposal of waste nitrogen at the expense of ureagenesis. Since nitrogen excretion via ammonium does not utilize any bicarbonate, but nitrogen excretion via urea does, the saved bicarbonate can neutralize excess protons.

As already mentioned, all of those mechanisms named above are valid for compensation of metabolic acidosis. In contrast, this is not true for respiratory acidosis, since both extracellular buffering, due to the inability of bicarbonate to buffer carbonic acid, and respiratory compensation are impossible [11]. In addition, intracellular buffering is not very effective in respiratory acidosis as can be seen from the Henderson-Hasselbalch equation (enlargement of the denominator). Therefore, the major proportion of excess protons must be compensated by the kidney or more specifically by an increase in ammonium excretion, as stated above.

In the present study, there is a trend showing that maximum values of ammonium excretion were reached in the presence of metabolic acidosis in most cases (Figure 3, “disturbance”). In contrast, in patients showing initial respiratory acidosis, values of excreted ammonium still increased after cessation of the initial disturbance (Figure 3, “compensation”). This may hint at a causal relationship between ammonium excretion and the subsequent increase in BE in this latter group. It explains why patients having initially suffered from metabolic acidosis in the postoperative course did not develop a BE > +2 mEq/L, whereas 80% of patients having had initial respiratory acidosis showed hereinafter a BE > +2 mEq/L (Figure 1). Due to the variability as regards individual ammonium baseline values, the patient's individual varying duration of the respective disturbances and the varying degree of acidosis, not the absolute values of ammonium excretion (Figure 3), are decisive, but rather the values over the course of time. The reason for this observed trend may lie in the number of compensatory mechanisms available for the respective disorder, which is reduced in the case of respiratory acidosis (see above), as well as the extent of the acid burden (see below), both resulting in a much stronger trigger in the case of respiratory acidosis compared to metabolic acidosis. The strength of this trigger may easily lead to an overcorrection in case of respiratory acidosis.

In addition to the named differences between respiratory and metabolic acidosis as regards compensation, there is one concerning the existing acid burden during the respective imbalance. The total buffer value of the whole body is defined as the sum of the buffering power *β* of the open bicarbonate system and the closed nonbicarbonate system [35]. Since *β*_nonbicarbonate_ changes relatively little over the clinically relevant range of acid-base disturbances, the total buffer value increases mainly as a function of plasma bicarbonate concentration. Therefore, at low plasma bicarbonate concentrations as in metabolic acidosis, the total buffer value is small, resulting in a much larger change of pH following administration of either protons or bicarbonate than when plasma bicarbonate concentration and hence total buffering value are high as in the case in respiratory acidosis. Therefore, after administration of a defined acid load, the concomitant change in pH is smaller in the case of respiratory acidosis, or, in other words, at a plasma pH value of the same magnitude, the acid burden is larger in respiratory compared to metabolic acidosis. Thus, in the event of respiratory acidosis, the need for compensation and subsequent correction is greater because of a larger acid burden. Since renal compensation is almost the only possibility for compensation in conditions of respiratory acidosis (see above) and renal compensation mainly consists of enhanced ammonium excretion, ammonium excretion must be strong. Due to a certain delayed onset of several hours of renal compensation, in contrast to the other compensatory mechanisms, a rebound with overshooting alkalemia is more likely in respiratory than in metabolic acidosis.

In the context of intraoperative acid-base status management, all patients received sodium bicarbonate by the perfusionist to antagonize dilutional acidosis originating from introduction of the priming and the cardioplegic solution into the patients' systemic circulation, which are both devoid of bicarbonate. Since all of the patients analyzed here received similar absolute amounts (Figure 4, Table 1), bicarbonate administration did not influence the main statement in the current study.

There are some limitations in the present study. The natural heterogeneity of the patient population with potential preexisting illnesses and individual ammonium baseline values, the varying duration of the observed disturbances in acid-base status, the varying degree of acidosis, and the different points of time at which the disturbance emerged in the postoperative interval lead to differences in the compensation's magnitude. But the intention of this observational study was not to evaluate the absolute amounts of excreted ammonium but rather to correlate the occurrence of increased ammonium excretion, possibly leading to a subsequent rise of BE > +2 mEq/L, with the acidosis origin. Furthermore, owing to the small number of patients, the validity might be limited. But the high significance of *p* < 0.01 obtained (Figure 1) nevertheless displays a strong force of expression, although we cannot completely exclude the possibility that the findings are due to type I statistical error. Future studies with larger patient cohorts are required.

## 5. Conclusions

In conclusion, the majority of patients who underwent CABG surgery displayed acidosis at the beginning of the postoperative period as regards imbalances in acid-base status. Strikingly, elevation of BE value above the upper limit of the reference range (i.e., > +2 mEq/L) did not occur after postoperative metabolic acidosis but after respiratory acidosis which could be correlated with maximum values of ammonium excretion, whereas in the case of metabolic acidosis, maximum ammonium values lay within the interval of the initial disturbance. Therefore, the acidosis origin seems to have a large influence on compensation and the possibility of an overcorrection. These data as part of basic research provided very useful insights into compensatory ammonium excretion which seems to depend on the acidosis origin.

## Figures and Tables

**Table 1:** Administered types of fluids and their respective volume during the operation as well as the postoperative phase dependent on the patients' initial postoperative disturbance in acid-base status.

	Patients with initial postoperative respiratory acidosis and subsequent BE > + 2 mEq/L		Patients with initial postoperative respiratory acidosis and no subsequent disorder		Patients with initial postoperative metabolic acidosis and no subsequent disorder	
	Intraoperative	Postoperative	Intraoperative	Postoperative	Intraoperative	Postoperative
Priming solution (0.9% NaCl) (ml)	1150 ± 100	—	1100	—	1125 ± 71	—
Bretschneider solution (ml)	1450 ± 332	—	1500	—	1600 ± 220	—
Sodium bicarbonate 8.4% (ml)	105 ± 47	—	100	—	106 ± 35	—
Jonosteril (ml)	2188 ± 688	—	3000	—	1813 ± 594	169 ± 477
Ringer's solution (ml)	—	2400 ± 387	—	950	—	2506 ± 937
0.9% NaCl (ml)	188 ± 149	1363 ± 622	—	350	225 ± 325	1363 ± 994
Gelafundin (ml)	338 ± 471	2250 ± 289	500	1500	450 ± 325	2375 ± 641
Red cell concentrate (ml)	75 ± 150	150 ± 300	—	—	75 ± 139	175 ± 388
